# Thymoquinone Induces Cell Death in Human Squamous Carcinoma Cells via Caspase Activation-Dependent Apoptosis and LC3-II Activation-Dependent Autophagy

**DOI:** 10.1371/journal.pone.0101579

**Published:** 2014-07-07

**Authors:** Shu-Chen Chu, Yih-Shou Hsieh, Cheng-Chia Yu, Yi-Yeh Lai, Pei-Ni Chen

**Affiliations:** 1 Institute and Department of Food Science, Central Taiwan University of Science and Technology, Taichung, Taiwan; 2 Institute of Oral Science, School of Dentistry, Chung Shan Medical University Hospital, Taichung, Taiwan; 3 Institute of Biochemistry and Biotechnology, Chung Shan Medical University, Taichung, Taiwan; 4 Clinical Laboratory, Chung Shan Medical University Hospital, Taichung, Taiwan; 5 Department of Dentistry, Chung Shan Medical University Hospital, Taichung, Taiwan; IISER-TVM, India

## Abstract

**Background:**

Thymoquinone (TQ), an active component of *Nigella sativa* or black cumin, elicits cytotoxic effects on various cancer cell lines. However, the anti-cancer effects of TQ on head and neck squamous cell carcinoma (HNSCC) remain unclear.

**Methodology/Principal Findings:**

In this study, TQ elicited a strong cytotoxic effect on SASVO3, a highly malignant HNSCC cell line. The mechanisms of this cytotoxic effect were concentration dependent. TQ also induced apoptotic cell death in SASVO3 cells as indicated by an increase in Bax expression and caspase-9 activation. Apoptosis was possibly caspase-9 dependent because the exposure of cells to a caspase-9 inhibitor partially prevented cell death. The exposed cells also showed increased levels of autophagic vacuoles and LC3-II proteins, which are specific autophagy markers. Cell viability assay results further revealed that bafilomycin-A1, an autophagy inhibitor, enhanced TQ cytotoxicity; by comparison, Annexin V and propidium-iodide staining assay results showed that this inhibitor did not promote apoptosis. TQ treatment also increased the accumulation of autophagosomes. Using a lentivirus-shRNA system for LC3 silencing, we found that cell viability was eradicated in autophagy-defective cells. An in vivo BALB/c nude mouse xenograft model further showed that TQ administered by oral gavage reduced tumor growth via induced autophagy and apoptosis.

**Conclusions:**

These findings indicated that TQ induced cell death in oral cancer cells via two distinct anti-neoplastic activities that can induce apoptosis and autophagy. Therefore, TQ is a promising candidate in phytochemical-based, mechanistic, and pathway-targeted cancer prevention strategies.

## Introduction

Cell death may occur in several mechanisms, including apoptosis, necrosis, and autophagy. Apoptosis is regulated by one of the two programmed, cellular signaling pathways, namely, death receptor-mediated pathway and mitochondrial pathway. Apoptotic cells are morphologically characterized by cell shrinkage, membrane blebbing, chromatin condensation, DNA fragmentation, apoptotic body formation, and caspase activation [1]. Autophagy is a catabolic process that maintains cellular homeostasis in response to various cellular stress factors, such as infection, nutrient starvation, protein aggregation, and organelle damage. Studies have also applied autophagy, another form of non-apoptotic cell death, as a form of cancer therapy [1], [2]. For instance, autophagy-inducing agents are used to treat cancer. In autophagy, double-membrane vesicles termed autophagosomes, which contain cytoplasmic components, are stimulated; these vesicles then fuse with lysosomes to form autolysosomes. In this process, degraded products are recycled in the cytoplasm. Low autophagy levels can promote cell survival, but high autophagy levels can cause catastrophic damage to a cell. As a result, autophagic cell death occurs. Studies have also shown that several anti-cancer drugs induce autophagic and apoptotic cell deaths in various cancer cells [2].

A diet rich in plant foods may help protect against cancer and reduce cancer risk because fruits, vegetables, flowers, whole grains, herbs, nuts, and seeds with significant terpenoids, sulfur and phenolic compounds, and other antioxidants are associated with cancer prevention and treatment [3], [4]. For instance, thymoquinone (TQ) is a phytochemical compound extracted from the plant *Nigella sativa* or black cumin, which is used extensively in Middle and Far Eastern countries as a spice and food preservative; TQ also exhibits medicinal effects, including anti-bacterial, anti-fungal, anti-viral, anti-inflammatory, immunomodulatory, and anti-cancer properties [5], [6]. Furthermore, TQ inhibits human cancer-cell proliferation and induces apoptosis [7]. Using a mouse xenograft model, [8] showed that the combined treatment of TQ and cisplatin is well tolerated; this treatment also significantly reduces tumor volume and weight without eliciting additional toxic effects on mice. TQ reduces tumor angiogenesis and growth by suppressing AKT and extracellular signal-regulated kinase signaling pathways [9]. Studies have revealed that TQ inhibits autophagy in glioblastoma cells by perturbing the lysosomal membrane and cathepsin translocation, resulting in caspase-independent apoptosis [10].

Studies on autophagy have focused on the degradation and induction mechanisms of various substances. For example, LC3 is present in two forms: (1) LC3I, which is cytosolic, and (2) LC3II, which binds to autophagosomes during autophagy. Therefore, LC3II expression is a widely investigated autophagy marker. However, western blot analysis results have revealed that LC3II is not significantly increased in a balanced autophagic flux [11]. This finding suggests that several autophagy inducers, including TQ, elicit an extremely imbalanced autophagic flux in cells and result in autophagosome accumulation. This study aimed to examine the function of autophagosome accumulation in TQ-mediated cell death in highly malignant oral squamous carcinoma cells. We demonstrated that the lysosome inhibitor bafilomycin-A1 (Baf A1) enhances TQ-induced autophagosome accumulation and autophagic cell death. TQ also induces apoptosis by activating caspase cascades in human oral SASVO3 cancer cells.

## Materials and Methods

### Cell culture

Dr. Cheng-Chia Yu (Taiwan, Chung Shan Medical University) generously provided the following cell lines: The Smulow–Glickman (S-G) human gingival epithelial cell line was original from F.H. Kasten, East Tennessee State University, Quillen College of Medicine, Johnson City, TN [12]; OC2 is a cell line derived from an oral squamous cell carcinoma specimen of a buccal-mucosa squamous carcinoma from a Chinese man. OC2 cell line was original from R.C. Chang [13]; SCC-4, a human tongue squamous cell carcinoma, was obtained from the Bioresource Collection and Research Center (Hsinchu, Taiwan) [14]; SAS, a high-grade tumorigenic human tongue squamous cell carcinoma, was obtained from the Japanese Collection of Research Bioresources (Tokyo, Japan) [15]; and SASVO3, a highly malignant head and neck squamous cell carcinoma (HNSCC) cell line obtained from primary SAS tumors using three sequential rounds of xenotransplantation [16]. SG, SAS, and SASVO3 cells were cultured in Dulbecco's modified Eagle's medium (DMEM) supplemented with 10% fetal bovine serum (FBS) and 2 mM glutamine. SCC-4 was cultured in DMEM supplemented with the nutrient mixture Ham's F-12 (Life Technologies, Grand Island, NY), 10% FBS, 2 mM glutamine, and 400 ng/mL hydrocortisone. OC2 was cultured in RPMI medium with 10% FBS in a humidified 5% CO_2_ atmosphere at 37°C.

### Cell viability (MTT assay) and cell growth assay

The cells were seeded onto 24-well plates at a density of 4×10^4^ cells/well for 24 h and then treated with TQ (0, 20, 40, and 60 µM) for another 24 h. Afterward, the cells were incubated with 0.5 mg/mL 3-(4,5-dimethylthiazol-2-y1)-2,5-diphenyltetrazolium bromide (MTT) in a culture medium for 4 h. The blue formazan crystals of viable cells were dissolved using isopropanol and then evaluated spectrophotometrically at 563 nm. For the cell growth assay, the treated cells were harvested and counted in duplicate by using a hemocytometer. Trypan blue exclusion assay was also performed to determine the viable and dead cells [17].

### Cell cycle analysis

At 24 h after cell seeding, SASVO3 cells were synchronized in a medium containing 8 mM glutamine and 0.04% FBS for another 24 h [18]. After synchronization, the cells were stimulated by adding a medium containing 10% serum and exposed to 0, 20, 40 and 60 µM TQ for 24 h. The cells were harvested, washed with phosphate-buffered saline (PBS), fixed in 70% ethanol for 2 h at −20°C, and stained with propidium iodide (PI) solution [25 µg/ml PI, 0.1 mM ethylenediaminetetraacetic acid (EDTA), and 10 µg/ml RNase in PBS] for 30 min in the dark. DNA content was measured using a FACScan laser flow cytometer analysis system (Becton Dickinson, San Jose, CA), and cells were analyzed in each experimental treatment.

### Western blot analysis

The cells were treated with TQ for 24 h, and the treated cells were lysed using a cold mammalian protein extraction buffer kit (GE Healthcare Bio-Sciences Corp., Piscataway, NJ) with protease inhibitor cocktails for 20 min to prepare the total cell lysates. The samples were separated in a 12.5% polyacrylamide gel and then transferred onto a nitrocellulose membrane. Afterward, the membranes were blocked in 5% non-fat milk in Tris-buffered saline with Tween buffer (20 mM Tris, 137 mM NaCl, pH 7.6, 0.1% Tween-20) for 1 h. The membranes were then probed with antibodies specific for caspase-9, caspase-8, Bax, Bcl-2, Bid (Santa Cruz Biotechnology Inc. California, USA), p-histone H2A.X, caspase-3 (Millipore Corp., Bedford, MA; Chemicon International, Inc., Temecula, CA), Rubicon, PI3K Class III, Becline, Atg14, Atg7, Atg16L1, Atg5, Atg12, LC3A, p62, PARP and mToR (Cell Signaling Technology Inc., Danvers, MA), along with appropriate peroxidase-conjugated secondary antibodies. The signal was subsequently detected using an ECL commercial kit, and relative photographic density was quantified using an ImageQuant LAS 4000 mini (GE Healthcare, Little Chalfont, Buckinghamshire, UK) [19].

### 4-6-Diamidino-2-phenylindole (DAPI) staining

The cells were treated with TQ for 24 h. The treated cells were then washed twice with PBS, fixed in 70% ethanol for 30 min at room temperature, and stained with DAPI (0.6 µg/mL PBS) for 5 min. Chromatin fluorescence was observed under a UV-light microscope, and the apoptotic cells were morphologically defined on the basis of cytoplasmic and nuclear shrinkage and chromatin condensation [19].

### Mitochondrial membrane potential (Δ*Ψ*m) assay

The cells were treated with TQ for 24 h. The treated cells were washed twice and incubated in a complete medium containing 10 µg of fluorescent lipophilic cationic JC-1 dye for 30 min at 37°C in the dark. The stained cells were harvested, washed, resuspended, and subjected to immediate flow cytometry analysis. JC-1 was selectively accumulated in the intact mitochondria to form multimer J-aggregates that emit fluorescence at 590 nm (red) with a high membrane potential. Monomeric JC-1 emits light at 527 nm (green) with a low membrane potential. Thus, the fluorescence color of JC-1 represents the mitochondrial membrane potential, which can be analyzed using a fluorescence-activated cell sorting system [20].

### Annexin V-FITC and PI staining assay

TQ-induced apoptotic SASVO3 death was quantified by flow cytometry with annexin V-FITC and PI staining. In brief, the cells were treated with TQ for 24 h. The treated cells were collected, washed twice with PBS, and subjected to annexin V and PI staining by using Vybrant Apoptosis Assay Kit 2 (Invitrogen, Carlsbad, CA) according to the manufacturer's step-by-step protocol. Recombinant annexin V conjugated to fluorophores and Alexa fluoro 488 dye provided maximum detection sensitivity. After staining, flow cytometry was performed to quantify apoptotic cells [20].

### Detection of autophagic vacuoles development

The autofluorescent substance monodansylcadaverine (MDC) has recently been shown to stain autophagic vacuoles. In brief, the treated cells were washed with Hank's buffered salt solution (HBSS) twice, stained with 1 µg/ml MDC (Sigma), and diluted in HBSS containing 5% FBS for 15 min. After staining, the cells were washed with HBSS, covered with HBSS containing 5% FBS, and observed under a green-filter fluorescence microscope.

### GFP-LC3 transfection and GFP-LC3 dot formation

LC3 cDNA was obtained from Addgene (Boston, MA). GFP-LC3 fusion protein was used to make the autophagosomes visible in cells. The cells were seeded onto 35 mm µ-Dish (ibidi, Germany). After overnight culture, cells were transfection with 3 µg GFP-LC3 expressing plasmid in each plate using PolyJet™ reagent (SignaGen Laboratories, Gaithersburg, MD) and incubated for 24 h. The medium was removed and fresh medium containing TQ with either with or without bafilomycin-A1 (Baf A1) was added to the plate for 24 h. At the end of treatment, cells were investigated by the EVOS FL (AMG Micro, Bothell, WA) microscope at 40× magnification [21].

### VZV-G pseudotyped lentivirus-short hairpin RNA system

RNAi reagents were obtained from the National RNAi Core Facility located at the Institute of Molecular Biology/Genomic Research Center in Academia Sinica; this study was supported by the National Research Program for Genomic Medicine Grants of the National Science Council [21].

### VZV-G pseudotyped lentivirus-shRNA production

Lentiviral infection was induced in SASVO3 cell line to integrate and express a stable shRNA targeting the *LC* mRNA sequences. Individual clones were identified on the basis of their corresponding unique RNAi consortium (TRC) numbers: shLuc TRCN0000072246 for vector control targeted to luciferase and shLC3 (91) TRCN0000243391 (responding sequence: AGC GAG TTG GTC AAG ATC ATC) targeted to *LC3* [21].

### Lentivirus-shRNA infection of cells

Approximately 5×10^5^ SASVO3 cells were subcultured in 60 mm plates. After 16 h of culture, the cells were infected with recombinant lentivirus vectors at a multiplicity of infection of 1. The medium was removed the following day, and the cells were selected by applying 5 µg/mL puromycin (Sigma, P8833) for 5 d [21].

### Caspase-9 activity assay

Caspase -9 activities were measured using the BioVision Caspase-9 Colorimetric Assay Kit (BioVision, CA). This assay is based on the principle that activated caspases in apoptotic cells cleave the synthetic substrates to release free chromophore *p*-nitroanilide (*p*NA), which is measured spectrophotometrically at 405 nm. The *p*NA was generated after specific action of caspase-9 on tertrapeptide substrates LEHD-*p*NA. The reaction mixture consisted of 50 µl cell lysate (100 µg), 50 µl 2× reaction buffer (containing 10 mM dithiothreitol) and 5 µl 4 mM LEHD-*p*NA substrate in a total volume of 105 µl. The reaction mixture was incubated at 37°C for 2 h and absorbance of the product was measured at 405 nm according to manufacturer's instruction [22].

### Bioluminescence imaging (BLI) measurement of tumor growth in nude mice

All of the procedures involving animals were conducted in accordance with the institutional animal welfare guidelines of the Institutional Animal Care and Use Committee (IACUC) of the Chung Shan Medical University (IACUC Approval Number: 1085). Four- to five-week old immunodeficient nude mice (BALB/c AnN.Cg*Foxn ^nu^*/Crl Narl mice) weighing 15 g to 17 g were used in the nude mouse xenograft model. The mice were housed in a pathogen-free environment at the Laboratory Animal Unit under a regular 12 h light/12 h dark cycle and provided ad libitum access to standard rodent chow (Laboratory Rodent Diet 5001, LabDiet, St. Louis, MO). SASVO3 cells were injected subcutaneously into the right front axilla of the mice (5×10^6^ cells/0.1 mL/mouse). At 10 d post-implantation, the mice were randomly separated into three groups (*N* = 5 for each group) and fed with olive oil (control) and TQ (10 and 25 mg/day/kg) suspended in olive oil via oral gavage. The average tumor volume at the beginning of treatment was approximately 82.95 mm^3^. BLI was performed using an IVIS50 animal imaging system (Xenogen Corp., Alameda, CA). Tumor growth was monitored on the basis of luciferase activity in SASVO3 cells; the photons emitted from the target site penetrated the mammalian tissue; these photons could be externally detected and quantified using a sensitive light imaging system [23].

### Immunohistochemistry analysis

Paraffin-embedded slides were deparaffinized, and antigen unmasking was carried out by microwave heating in citrate buffer for 20 min. Slides were incubated with primary anti-Ki67 antibodies and biotinylated secondary anti-mouse antibodies were added [24].

### Statistical analysis

Statistical significances were analyzed by one-way analysis of variance (ANOVA) with post hoc Dunnett's test. *P* value <0.05 was considered statistically significant (Sigma-Stat 2.0, Jandel Scientific, San Rafael, CA).

## Results

### TQ potently reduces viability in oral cancer cells

**Figure 1:**
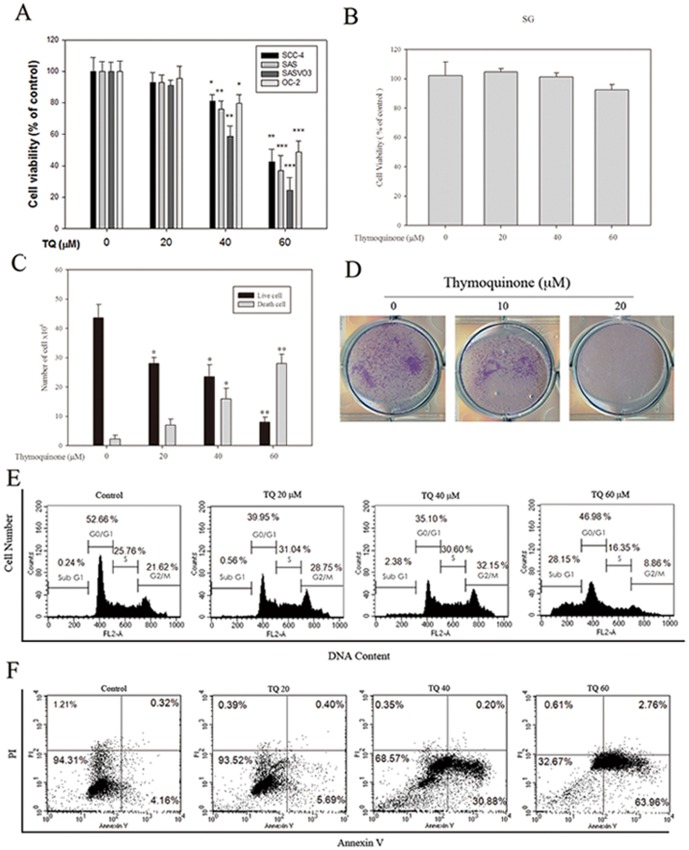
Effect of TQ on cell viability and apoptosis in oral cancer cells. (A) SCC-4, SAS, SASVO3, OC2, and (B) S-G cells were treated with various TQ concentrations for 24 h. Cell viability was analyzed by MTT assay. (C) SASVO3 cells were treated with TQ for 24 h. Viable cells were then collected and counted using a hemocytometer. (D) Equal numbers of SASVO3 cells were plated and stained using colony formation assay as described in the text. (E) SASVO3 cells were treated with TQ (0, 20, 40, and 60 µM), and cell cycle distributions were assessed by flow cytometry using PI staining. (F) Flow cytometry analysis of annexin V/PI double staining was conducted to determine the number of apoptotic cells. The statistical significance of the results was analyzed by one-way ANOVA and post hoc Dunnett's test (^*^ *p*<0.05, ^**^ *p*<0.01, ^***^ *p*<0.001).

MTT assay results showed that TQ strongly inhibited the growth of various oral cancer cell lines (**Figure 1A**). The IC_50_ concentrations in the SCC-4, SAS, SASVO3, and OC2 cells were 56.02, 53.33, 45.02, and 59.13 µM, respectively. The results showed that the 24 h treatment with TQ at various concentrations elicited significant concentration-dependent cytotoxic effects on these cells. No significant inhibitory effects were observed in a normal human gingival epithelial S-G cell (**Figure 1B**). Among the four oral cancer cell lines, SASVO3 cells were affected at the highest extent. TQ reacted with trypsin-EDTA and exhibited the strongest reduction effect on the number of viable SASVO3 cells in a dose-dependent manner (*p*<0.01; **Figure 1C**). To further investigate the anti-cancer activity of TQ, we performed a colonogenic assay and determined the long-term effect of TQ-induced autophagy on lung cancer cells. TQ significantly inhibited SASVO3 cell colony formation (**Figure 1D**).

### TQ induces apoptosis in SASVO3 cells

**Figure 2:**
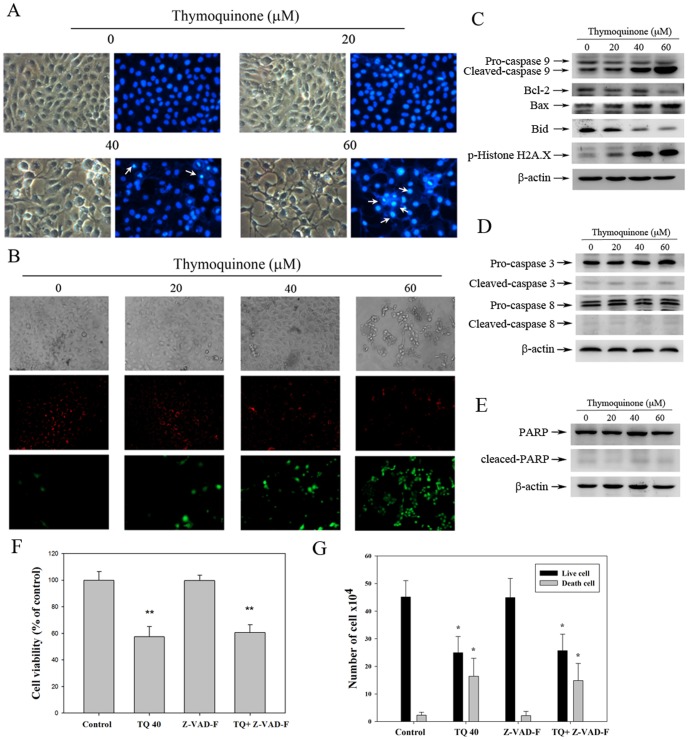
Apoptotic patterns of SASVO3 cells treated with TQ. Cells cultured with various TQ concentrations for 24 h were examined for apoptosis. (A) Cells were stained with DAPI and observed under a UV-light microscope to examine the nuclear morphology of the SASVO3 cells. The arrows show the areas with intense fluorescence staining and condensed nuclei (at a magnification of 200×). (B) Changes in Δ*Ψ*m were assessed using fluorescent lipophilic cationic JC-1 dye. JC-1 is selectively accumulated in intact mitochondria to form multimer J-aggregates that emit fluorescence at 590 nm (red) with a high membrane potential, *upper*. Monomeric JC-1 emits light at 527 nm (green) with a low membrane potential, *lower*. Western blot analysis was conducted on (C) caspase-9, Bcl-2, Bax, Bid, p-Histone H2A.X, (D) caspase-3, caspase-8, and (E) PARP. β-actin was used as a loading control. A result representing three separate experiments is shown.

PI staining was used to analyze the effect of TQ on the cell cycle and examine whether or not the inhibition of TQ-induced cell proliferation is associated with cell cycle arrest. The population of cells in the sub G1 phase (apoptotic cell population) increased (**Figure 1E**). The results of Annexin V/PI staining (**Figure 1F**) showed a significant increase in Annexin V staining (increased percentage of cell population in the lower right quadrant) from 4.16% to 63.96%. To further investigate whether or not apoptosis was induced by TQ in SASVO3 cells, DAPI staining was used to analyze the nuclear morphology of cells after 24 h of TQ treatment. The result showed that chromatin condensed in TQ-treated cells (**Figure 2A**). We then investigated the effects of TQ on mitochondrial permeability to examine whether or not the increase in mitochondrial disruption may account for the apoptotic effect of TQ. Δ*Ψ*m of the mitochondria was depolarized when SASVO3 cells were exposed to TQ; this result was indicated by a decrease in red fluorescence and an increase in green fluorescence (**Figure 2B**). Bcl-2 family proteins perform important functions in the regulation of apoptosis by acting as either promoters (e.g., Bax) or inhibitors (e.g., Bcl-2) of cell death; this mechanism may induce caspase activation. Western blot analysis results revealed the effect of TQ on the expression of these proteins in SASVO3 cells. In particular, TQ significantly increased Bax expression; however, the levels of Bcl-2 and Bid proteins were reduced in a dose-dependent manner (**Figure 2C**). By comparison, active caspase-9 was increased significantly in TQ-treated SASVO3 cells (**Figure 2C**). To investigate whether or not the induction of apoptosis was correlated with the induction of DNA damage, we examined histone H2A.X phosphorylation, which is an early cellular response to DNA damage [25]. The results showed that TQ induced H2A.X phosphorylation in a dose-dependent manner (**Figure 2C**), whereas it has no significant effect on cleaved-caspase 3, cleaved-caspase 8 (**Figure 2D**) and cleaved-PARP expression (**Figure 2E**). Further studies were performed to delineate the role of caspases activation in TQ-caused apoptosis of SASVO3 cells. Cells were pre-treated with pan-caspase inhibitor (CI, 50 µM Z-VAD-FMK) for 2 hours, and followed by TQ (40 µM) treatment for 24 h. Thereafter, cells viability and the number of dead cells were analyzed for MTT and trypan blue exclusion assay, respectively. In the presence of Z-VAD-FMK, it has no significant effect on TQ-induced cell death (**Figure 2F & 2G**). These results were further confirmed by caspase-3 activation assay. Preincubation of cells with Z-VAD-FMK resulted in a significant reduction of caspase-3 activity in SASVO3 cells (data not shown). Taken together, the results indicate that TQ-induced apoptosis in SASVO3 cells is not mediated through the activation of caspase-3.

### TQ induces autophagy in SASVO3 cells

**Figure 3:**
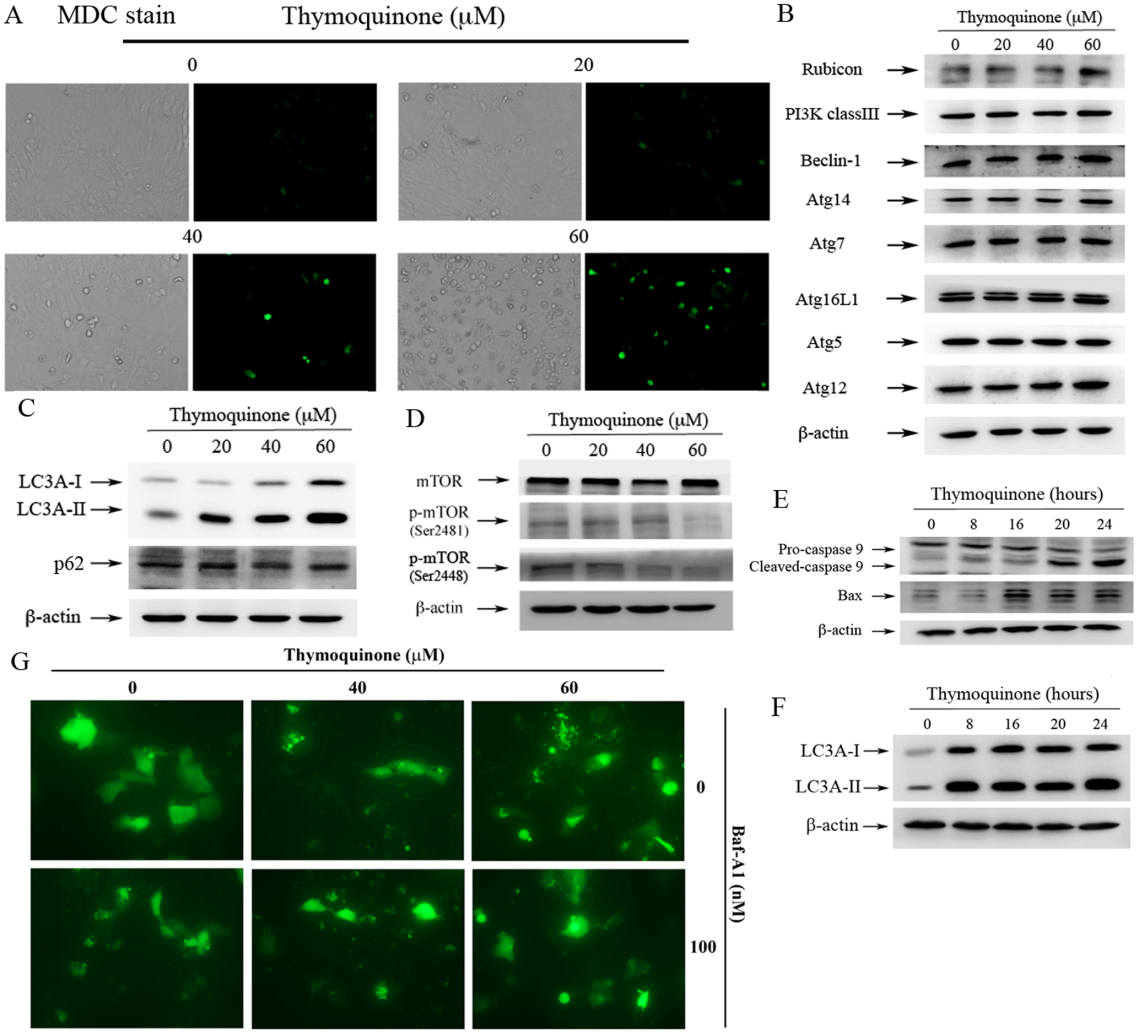
TQ induces autophagy in SASVO3 cells. (A) Cells were treated with TQ for 24 h and then stained with MDC visualized at magnification of 200× under a fluorescence microscope. Cells were treated with TQ for 24 h and Western blot analysis was performed to evaluate (B) autophagy-related protein, (C) LC3-I, LC3II, p62, (D) total mTOR, and p-mTOR (Ser2481 and Ser2448). β-actin was used as an internal control. Cells were treated with TQ for an indicated period of time, and Western blot analysis was conducted on (E) cleaved caspase-9, Bax, (F) LC3A-I, and, LC3AII. β-actin was used as a loading control. (G) SASVO3/GFP-LC3 cells were treated with 100 nM bafilomycin-A1 (Baf A1) and TQ (40 and 60 µM) for 24 h. The GFP-LC3 dots induced by TQ and bafilomycin-A1 in SASVO3/GFP-LC3 cells were observed. A result representing three separate experiments is shown.

Studies have shown that cancer cells undergo autophagy in response to various anticancer therapies. To investigate whether or not autophagy was involved in TQ-induced cell death, we conducted monodansylcadaverine (MDC) staining. The autofluorescent substance MDC has been used as a specific marker of autophagic vacuoles. Autophagic vacuoles distributed in the cytoplasm or perinuclear regions appear as distinct dot-like structures when these vacuoles are stained with MDC. The results of the present study showed that the number of MDC-labeled vesicles in TQ-treated cells increased in a dose-dependent manner compared with that in the control cells. This finding suggested that autophagy was induced after TQ treatment (**Figure 3A**). Western blot analysis was then conducted to determine autophagy-related protein expression. We confirmed that Beclin-1, Class III PI3K complex, Rubicon, and Atg family proteins are involved in autophagosome formation and autophagy initiation. Once autophagy is induced, LC3-I is directly conjugated to the lipid phosphatidylethanollamine and inserted into autophagic membranes to produce LC3-II, a protein marker of autophagy. mTOR is an important negative regulator of autophagy. Rubicon, Beclin-1, and Atg 14 expressions were increased slightly at 60 µM TQ (**Figure 3B**). An increase in LC3A-II was observed after the cells were treated with TQ, whereas it has no significant effect on p62 expression (**Figure 3C**). The phosphorylation of mTOR (Ser2481 and Ser2448) was also downregulated in a dose-dependent manner (**Figure 3D**). These data indicated that mTOR is involved in TQ-induced autophagy. Our data further showed that TQ induces apoptosis and autophagy in SASVO3 cells. Western blot analysis was also performed to examine the apoptosis-related proteins (caspase-9 and Bax) and autophagy marker (LC3A-II) at different times and determine the time sequence between apoptosis and autophagy. The results showed that an increase in LC3A-II was observed after 8 h (**Figure 3F**), and Bax was increased after 16 h. Cleaved caspase-9 was detected 20 h after the cells were treated with TQ (**Figure 3E**). Using a microscope, autophagosome accumulation was markedly enhanced after co-treatment with TQ and bafilomycin-A1 in SASCO3/GFP-LC3 cells (**Figure 3G**).

### TQ induced cell death in oral cancer cells via two distinct pathways with the capability to induce apoptosis and autophagy

**Figure 4:**
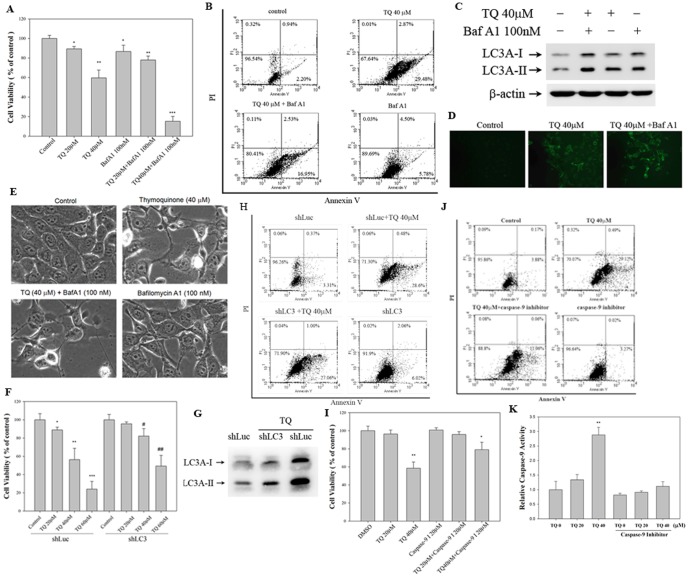
TQ induces cell death in SASVO3 cells via two distinct pathways that can induce apoptosis and autophagy. (A) Cells were pre-treated with Baf A1 (autophagy inhibitor) for 1 h and then exposed to TQ (20 and 40 µM) for 24 h. Cell viability was analyzed by MTT assay. (B) Cells were pre-treated with Baf A1 for 1 h and/or TQ for 24 h. Flow cytometry analysis of annexin V/PI double staining was performed to determine the number of apoptotic cells. (C) Western blot analysis was conducted on LC3-I and LC3-II, and β-actin was used as a loading control. (D) TQ and/or Baf A1 treatment increased the number of MDC-labeled vesicles. (E) Cytoplasmic vacuolization was observed in SASVO3 cells after Baf A1 and/or TQ treatment. (F) TQ-mediated cytotoxicity was analyzed in SASVO3 shLuc (vector control) and SASVO3 shLC3 cells by MTT assay. (G) Expression of LC3A-I and LC3A-II were analyzed in SASVO3 shLuc and SASVO3 shLC3 cells by Western Blot. (H) TQ-mediated apoptosis was analyzed in SASVO3 shLuc and SASVO3 shLC3 cells by flow cytometry analysis. (I) Cells were pre-treated with caspase-9 I (caspase-9 inhibitor) for 1 h and then exposed to TQ (20 and 40 µM) for 24 h. Cell viability was analyzed by MTT assay. (J) Cells were pre-treated with caspase-9 inhibitor for 1 h and then exposed to TQ (40 µM) for 24 h. The number of apoptotic cells was analyzed by flow cytometry. (K) Caspase-9 activity was measured using a caspase-9 activity assay kit. The statistical significance of the results was analyzed using one-way ANOVA with post hoc Dunnett's test (^*^ *p*<0.05, ^**^ *p*<0.01, ^***^ *p*<0.001; ^#^ *p*<0.05, ^##^ *p*<0.01).

To reveal the mechanism by which the relationship of these processes contributed to TQ-induced cell death, we exposed the cells to the apoptosis inhibitor caspase-9 inhibitor (caspase-9 I) and the autophagy inhibitor Baf A1. Conducting a cell viability assay, we found that Baf A1 enhanced TQ-induced cytotoxicity (**Figure 4A**). The results of annexin V/PI staining showed that the population of TQ-induced apoptotic cells decreased from 29.48% to 16.95% after the cells were treated with Baf A1 (**Figure 4B**). We also examined the autophagic flux by using Baf A1, which is an inhibitor of autophagosomal lysosome degradation and promotes LC3II protein accumulation. Baf A1-pre-treated SASVO3 cells exhibited an increase in LC3II accumulation (**Figure 4C**) and increase in the number of MDC-labeled vesicles (**Figure 4D**). We also examined the morphological changes in the cells after Baf A1 and/or TQ treatment. The cells treated with Baf A1 and/or TQ revealed cytoplasmic vacuoles that were potential autophagosomes, compared with the control cells (**Figure 4E**). TQ-mediated cell viability was observed in SASVO3 shLuc (vector control) and shLC3 cells. LC3 silencing prevented the TQ-induced cell death of SASVO3 cells (**Figure 4F**). In SASVO3 shLC3 cells, TQ-induced LC3A expression was blocked (**Figure 4G**). In comparison with SASVO3 shLC3 cells, TQ-caused apoptosis has no significant effect in SASVO3 shLC3 cells by flow cytometry (**Figure 4H**). SASVO3 cells were incubated with TQ either with or without a caspase-9 inhibitor for 24 h to evaluate the contribution of caspase-9 activity to TQ-mediated cell death. The presence of a caspase-9 inhibitor slightly increased the percentage of viable TQ-treated cells compared with the percentage obtained in cells treated with TQ alone (**Figure 4I**). Thereafter, cells were harvested for flow cytometry analysis of annexin V-stained cells. The results of annexin V/PI staining showed that the population of TQ-induced apoptotic cells decreased from 29.12% to 11.06% after the cells was treated with caspase-9 inhibitor (**Figure 4J**). Caspase-9 activity was confirmed using a caspase-9 activity assay kit. Preincubation of cells with caspase-9 inhibitor resulted in a significant reduction of caspase-9 activity in SASVO3 cells (**Figure 4K**).

### Anti-tumor effects of TQ in vivo

**Figure 5:**
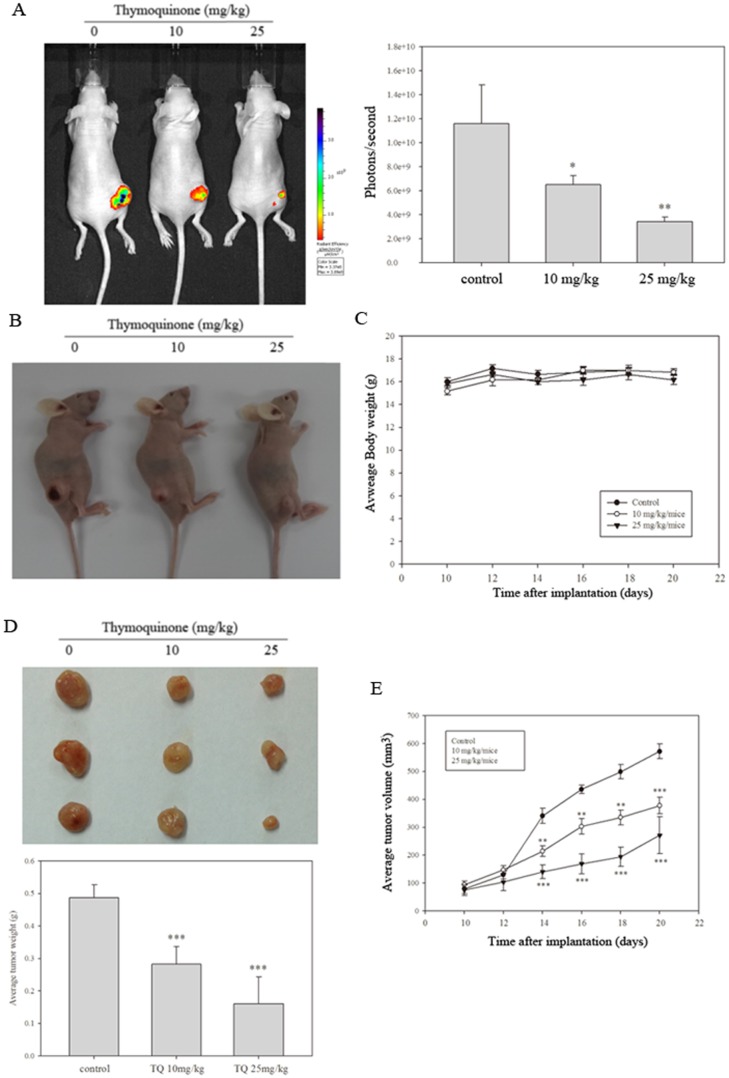
TQ elicits in vivo anti-tumor effects. BALB/c nude mice (*N* = 5 for each group) were treated with either olive oil or TQ after SASVO3 cells were subcutaneously implanted (s.c). Tumor growth was then analyzed. (A) Bioluminescence over time after s.c. SASVO3-cell inoculation. (B) Morphological characteristics of the control group treated with 10 and 25 mg/kg/day TQ. (C) Average body weight of the mice. (D) Average tumor weight and (E) tumor volume. Shown are means and standard errors, and the results were statistically evaluated using one-way ANOVA with post hoc Dunnett's test (^*^ *p*<0.05, ^**^ *p*<0.01, ^***^ *p*<0.001).

**Figure 6:**
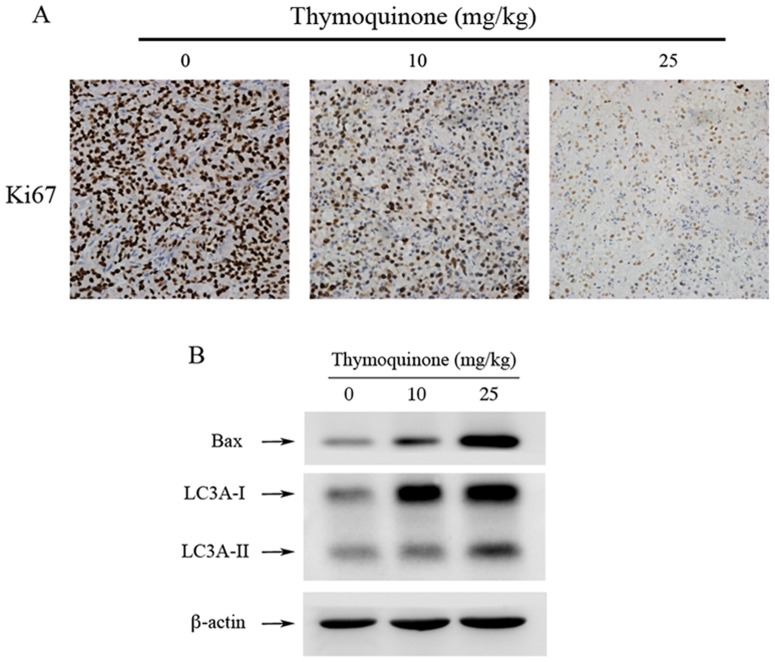
TQ reduces tumor growth and induces apoptosis and autophagy in vivo. (A) Ki-67 (cell proliferation marker) immunohistochemistry in SASVO3 tumors. (B) Bax level and LC3 expression and conversion in SAS tumors were determined by western blot analysis. β-actin was used as a loading control.

Luciferase-expressing and SASVO3-bearing nude mice were treated with either olive oil or TQ to verify the in vivo antitumor effects of TQ. Luciferase expression indicated that reactive ion etching significantly reduced tumor growth (*P*<0.01) without manifesting signs of toxicity (**Figures 5A and 5B**); this result was further confirmed by our data obtained by body weight monitoring (**Figure 5C**) throughout the experiment. TQ treatment reduced tumor weight (**Figure 5D**) and volume of the nude mice (**Figure 5E**) at 20 d compared with those of the control mice. TQ (25 mg/kg) treatment also induced a 3.04-fold reduction in tumor weight at 20 d (**Figure 5D**). Small solid tumors were also observed at 10 d after cell inoculation; 1.51- and 2.10-fold decrease in TQ-treated (10 mg/kg and 25 mg/kg) mice were observed at 20 d compared with that in the control mice (**Figure 5E**). A significant increase in proliferation determined by Ki-67 tumor staining was observed, and this result is consistent with the profound effect on tumor size (**Figure 6A**). The results of western blot analyses of these tumors revealed that TQ-treated tumors exhibited higher Bax and LC3II levels than those of the control SASVO3 tumors (**Figure 6B**). These data also suggested that TQ treatment induced apoptosis and autophagy in vivo.

**Figure 7:**
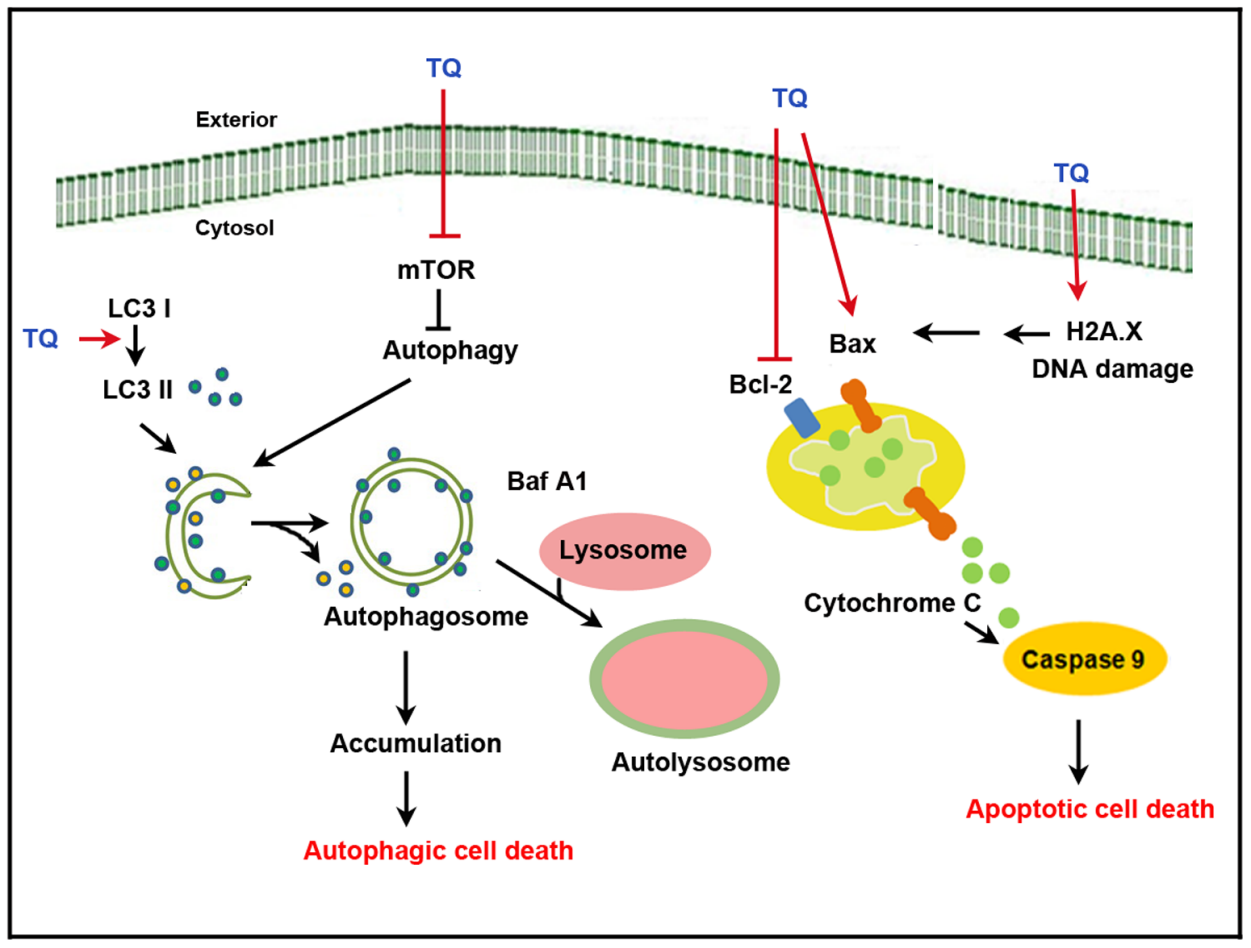
Proposed molecular targets in TQ anti-cancer efficacy in SASVO3 cells. TQ induced cell death in SASVO3 cells via two distinct antineoplastic activities that can induce apoptosis and autophagy. TQ induced autophagosome accumulation, resulting in autophagic cell death and increased DNA damage, potential Δ*Ψ*m collapse, chromosome condensation, and caspase-9 activation-dependent apoptosis.

In summary, TQ induced cell death in oral SASVO3 cancer cells in two distinct anti-neoplastic activities that can induce apoptosis and autophagy via caspase-activation-dependent apoptosis and LC3-II-activation-dependent autophagy (**Figure 7**).

## Discussion

HNSCC is one of the most common cancers in the world and exhibits an overall five-year survival rate of approximately 50%. HNSCC is less dependent on staging or lesion sites compared with other cancer types [26]. Death caused by oral cancer is often the result of local recurrence and regional or systemic metastasis. Despite the advancements in the diagnosis and management of HNSCC, long-term survival rates improved only marginally in recent decades [27]. Hence, novel candidate compounds should be developed and the molecular mechanisms of carcinogenesis should be understood to develop therapeutic approaches for patients with HNSCC. An alternative approach to cancer progression decline may promote chemoprevention. One approach in advanced oral cancer management involves the use of plant products, which are also known as nutraceuticals. These natural compounds entail relatively low cost and elicit non-toxic effects; these compounds are also physiologically bioavailable and have multiple molecular targets in cancer chemoprevention [4], [23]. Natural products have been extensively investigated in the prevention or intervention of tumorigenesis and neoplastic progression in early stages prior to the onset of invasive malignant diseases [28]. However, several effective cancer chemotherapeutic drugs, such as doxorubicin and cisplatin, exhibit major drawbacks, including drug resistance and systemic toxicity. To overcome such problems, researchers conducted studies on combination chemotherapy focusing on natural compounds with known active mechanisms that can increase the therapeutic index of clinical anticancer drugs [29]. Extensive studies on TQ have also shown promising anti-cancer effects. In the present study, TQ could be potentially used to prevent tumor growth in vivo and induce apoptotic and autophagic human oral cancer cell death. A recent study has shown that allometric scaling is an empirical method for predicting anatomical, physiological, and pharmacokinetic measures across species in relation to size and time [30], [31]. The conversion factor (*km*) is simply the body weight divided by the body surface area. Thus, by using *km* factors, the dose in Species 1 (in mg/kg) is equivalent to (*km*_species2_/*km*_species1_) times the dose in Species 2 (in mg/kg). In our study, 10 mg/kg and 25 mg/kg doses of thymoquinone in mice would be equivalent to 0.83 mg/kg and 2.08 mg/kg doses in human, respectively.

Apoptosis functions in the prevention of cancer progression; however, the function of autophagy remains unclear. Furthermore, autophagy is a eukaryotic process and functions in starvation, cell death, cell survival, aging, and tumor prevention [1], [2]. The survival functions of autophagy suggest that this response may contribute to tumor development. Autophagic regulation may also enhance anti-cancer therapeutics. However, evidence has suggested that overstimulated autophagy can result in autophagic cell death. The pro-survival and pro-death functions of autophagy remain unclear because this mechanism is dependent on cell type and stress level. If inhibited, autophagy can sensitize tumor cells in response to treatment by stimulating autophagic cells or activating apoptosis. Autophagy is characterized by autophagosome formation; these autophagosomes then fuse with lysosomes to form autolysosomes. In the present study, autophagic vacuole formation was determined by punctuate MDC staining. Baf A1 enhanced TQ-induced cytotoxicity; the accumulation of LC3II and the number of MDC-labeled vesicles were also increased. Apoptosis and associated cellular events exhibit profound effects on the progression of cancer cells from the benign to the malignant phenotype; such events are an ideal target in the therapy of various cancer cells [32]. Apoptosis assays further demonstrated that TQ treatment induced caspase-9 activation, chromatin condensation, and apoptotic SASVO3 cell death. Our data also suggested that TQ may induce SASVO3 cell death not only by caspase-activation-dependent apoptosis but also by autophagy.

Reactive oxygen species (ROS), which is a collective term for oxygen-derived species, including superoxide anion radical (O^2·−^), hydroxyl radicals (·OH), and hydrogen peroxide (H_2_O_2_), are initially considered as normal by-products of cellular metabolism [33]. Studies have shown that ROS regulate many cellular responses, including apoptosis and autophagy, via the mitochondrial signaling pathway. These data have also suggested that various cancer cells are associated with increased metabolic activity and ROS production under oxidative stress. Thus, cancer cells are vulnerable to damage caused by ROS because scavenging increased ROS induced by exogenous agents or drugs in cancer cells is more difficult than that in normal cells [34]. Hence, an exogenous ROS inducer can selectively cause cancer cell death by disrupting the mitochondrial membrane and activating numerous pathways to trigger apoptosis [35]. ROS can also function as signaling molecules to modulate autophagy [36]. The loss of TP53-inducible glycolysis and apoptosis regulator enhances ROS-dependent apoptosis [37] modulates ROS in response to nutrient starvation or metabolic stress; this condition also inhibits autophagy [36]. Hirsutanol A elicits autophagy and apoptosis by ROS accumulation; human breast cancer MCF-7 cells can be sensitized to hirsutanol A when these cells were co-treated with Baf A1, which is an autophagy inhibitor [38]. In the present study, TQ treatment inhibited ROS formation in SASVO3 cells (data not shown).

Studies have confirmed a crosstalk between autophagy and apoptosis [39]. However, other studies have indicated that autophagy inhibits apoptosis [40]. Autophagy also functions in induction of autophagic cell death [21]. PUMA, Bax, and active caspase-8 indicate that AJ-5, a novel binuclear palladacycle complex, activates intrinsic and extrinsic apoptotic pathways. AJ-5 treatment also simultaneously induces autophagosome formation, resulting in autophagic cell death. These findings are similar to those in our study. In particular, the combined treatment with TQ not only induced apoptosis but also elicited autophagic cell death in human oral cancer cells. Studies on glioblastoma cancer cells have indicated that TQ inhibits autophagy by perturbing the lysosomal membrane and cathepsin translocation from the lysosome lumen to the cytosol. Caspase-independent apoptotic cell death is then induced [10]. Sodium selenite induces apoptosis by generating ROS; Baf A1 pre-treatment enhances sodium selenite-induced apoptosis; this result indicated that sodium selenite-induced autophagy functions as a survival mechanism in human lung A549 cancer cells [40]. Studies on gastric cancer cells have revealed that akebia saponin induces autophagy-promoted, mitogen-activated, and protein kinase-mediated apoptosis [41]. As an AMP-activated protein kinase (AMPK) inhibitor, compound C suppresses cell proliferation by inducing apoptosis and autophagy in human colorectal cancer cells, and the preferred death pathway is cell type dependent [42]. These data indicate that the effects of an autophagy inducer or inhibitor on either caspase-dependent or caspase-independent apoptotic cell death may depend on cell type. In the current study, the results of Annexin V/PI staining (**Figure 1F**) showed a significant increase in Annexin V staining from 4.16% to 63.96%, while the presence of a caspase-9 inhibitor slightly increased the percentage of viable TQ-treated cells compared with the percentage obtained in cells treated with TQ alone from 58.54% to 79.10% (**Figure 4I**). We cannot exclude the possibility that there may be a cross-talk between autophagy and apoptosis by treatment thymoquinone in SASVO3 cells.

TQ has recently been applied as a promising compound in either the prevention or treatment of malignant tumors. Given the molecular complexity of the signaling pathways involved in cancer development and progression, multiple target therapy may be considered as a successful strategy in anti-cancer treatment. In conclusion, a novel mechanism of TQ involves the induction of autophagosome accumulation, resulting in autophagic cell death and caspase-9 activation-dependent apoptosis.
